# Melanotic neuroectodermal tumor of infancy: a case report

**DOI:** 10.1186/s13256-024-04550-y

**Published:** 2024-05-02

**Authors:** Mesfin Asefa, Tedros Hailu

**Affiliations:** 1 Department of Pathology, St. Paul’s Hospital Millennium Medical College (SPHMMC), Addis Ababa, Ethiopia; 2 Department of Pediatrics, Medical Faculty, Tikur Anbesa Specialized Hospital, Addis Ababa University, Addis Ababa, Ethiopia

**Keywords:** Melanotic, Neuroectodermal, Tumor of infancy, Case report

## Abstract

**Background:**

Melanotic neuroectodermal tumor of infancy (MNTI) is a rare clinically benign, pigmented, tumor of neural crest origin which commonly occurs in the maxilla. It is a rare tumor that may pose difficulty in differentiating from other malignant round cell tumors.

**Case presentation:**

A 5-month-old Ethiopian infant presented with a mass on his forehead. A wide excision of the lesion was done and subjected to histopathologic evaluation. The histologic and immunohistochemistry for synaptophysin studies confirmed that the infant was having MNTI. The patient was followed and there was no sign of recurrence at the 6th and 9th months of follow-up.

**Conclusion:**

MNTI should be considered as a differential diagnosis for tumors occurring in the head region in infants and prolonged follow-up may be needed to check for possible recurrence of the tumor.

## Introduction

MNTI is a rare clinically benign tumor in infants that needs to be differentiated from other malignant childhood tumors like neuroblastoma and alveolar rhabdomyosarcoma [1, 2]. It commonly occurs in the alveolar ridges of the maxilla and less commonly in the skull and extremities [3]. Computed tomography (CT) and FNA are the choices of initial diagnosis. A histopathologic examination can confirm the diagnosis based on the findings of the two cell populations; small round cells and larger melanocyte-like cells containing dark brown pigment in a fibrillary and collagenized vascular stromal background. The treatment is wide-margin excision and follow-up for a possibility of recurrence and malignant transformation. A classic, rare, case of melanotic neuroectodermal tumor of infancy is presented and its clinical, radiological, pathological, therapeutic, and prognostic aspects are discussed.

### Patient information

A 5-month-old Ethiopian male infant presented with a firm, non-tender mass on the forehead at the midline. The swelling was initially noticed at the age of one month by his mother which progressively increased in size. The child was brought to Tikur Anbesa Specialized Teaching Hospital, Addis Ababa, to the Department of Pediatrics. The pregnancy and delivery had been normal and there was no history of medication during pregnancy. The growth and development of the neonate were adequate for his age. There was not any other mass identified on physical examination and by abdominal ultrasound.

### Clinical findings and diagnostic assessment

**Fig. 1:**
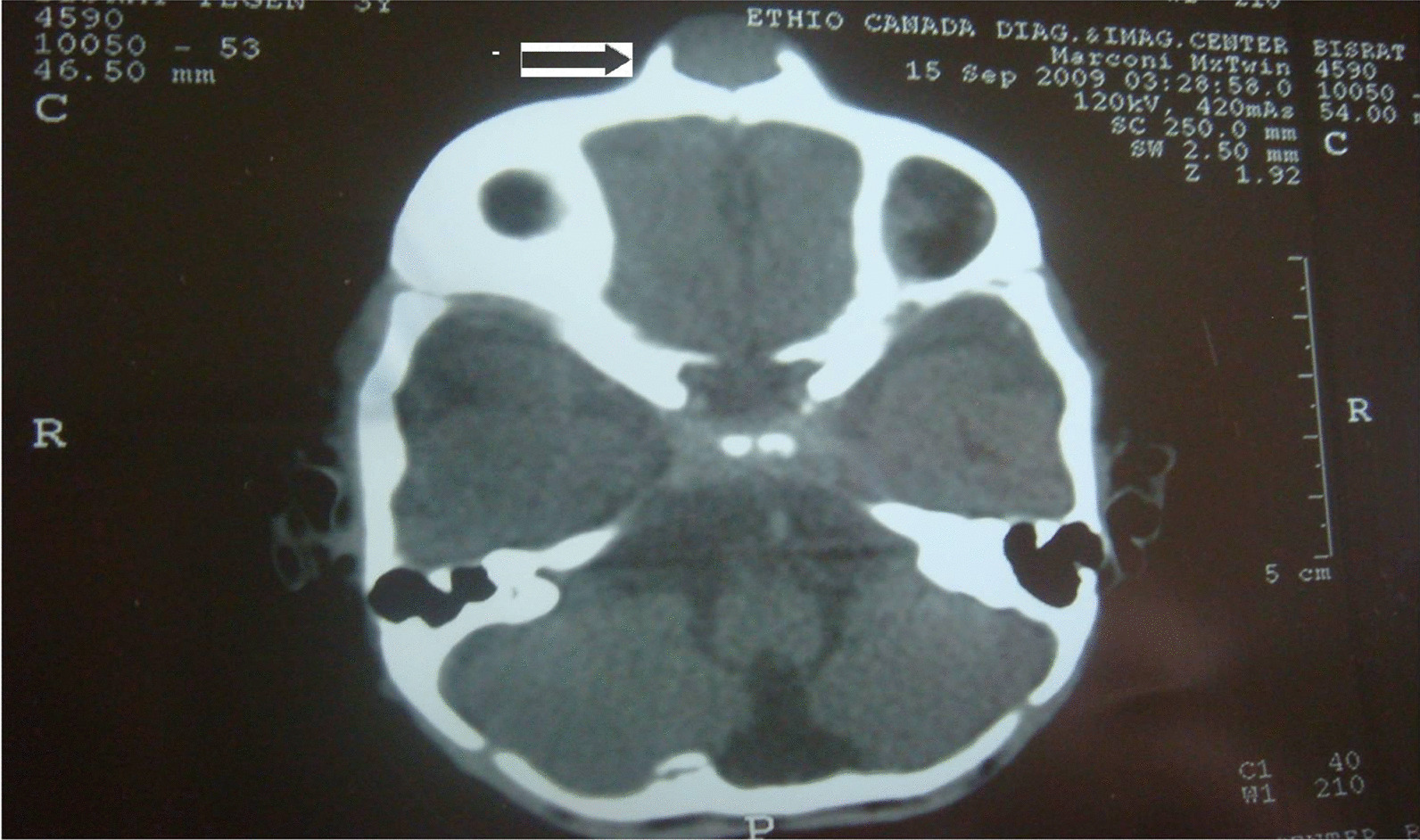
Computed tomography showing forehead mass with peripheral bone cuffing (hyperostostic) (arrow)

The smooth swelling measures 6 × 6 cm. The overlying skin was intact but stretched. No pulse/thrill was elicited. On palpation it was firm to hard, non-tender and non-fluctuant. The results of routine laboratory studies were within normal limits (urinary vanillylmandelic acid was not possible to determine). Plain radiographs of the skull (posteroanterior and lateral projection) and plain and contrast computed tomography (CT) of the skull were taken. The skull radiographs revealed a soft tissue swelling with sclerosis of the underlying frontal bone along the midline. Plain and contrast computed tomography (CT) of the skull showed a midline frontal (16 × 16 mm) cystic mass with peripheral reactive bone cuffing (hyperostosis) but no underlying bone defect. Dermoid cyst was entertained radiologically (Fig. 1). FNAC from the mass revealed small round (Neuroblast-like) cells and reported as small round cell tumor with a suggestion for biopsy confirmation.

### Therapeutic intervention

At the age of 5 months, wide-based excision and curettage of the area down to the normal bone were done under general anesthesia. The infant tolerated the anesthetic and surgical procedures well and was discharged in good condition on the 6th postoperative day. The removed mass was subjected to histopathologic examination.

### Follow-up and outcomes

**Fig. 2:**
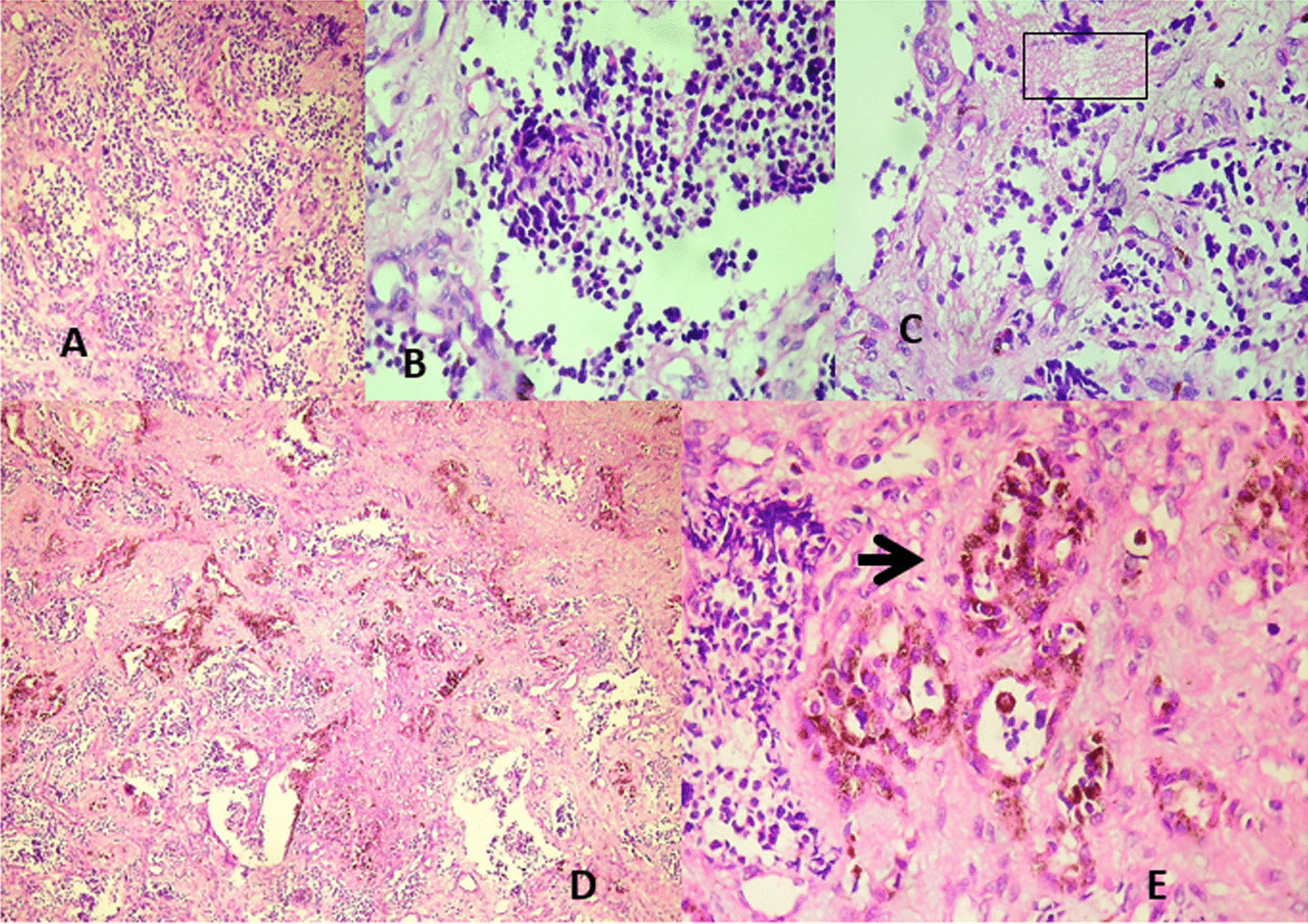
Predominant small neuroblast-like cells in a collagenized stroma (**A**) (Haematoxylin & Eosin stain(H&E), × 20); High power (H&E, × 40) view of the small round neuroblast-like cells (**B**); The small round cells in a neurofibrillary background, ( window) (**C**); Small round cells & larger pigmented cells in alveolar patterns in a collagenized stromal background (**D**) (H&E, × 10); Larger pigmented cells in a pseudoglandular pattern (arrow) (**E**) (H&E, × 40)

**Fig. 3:**
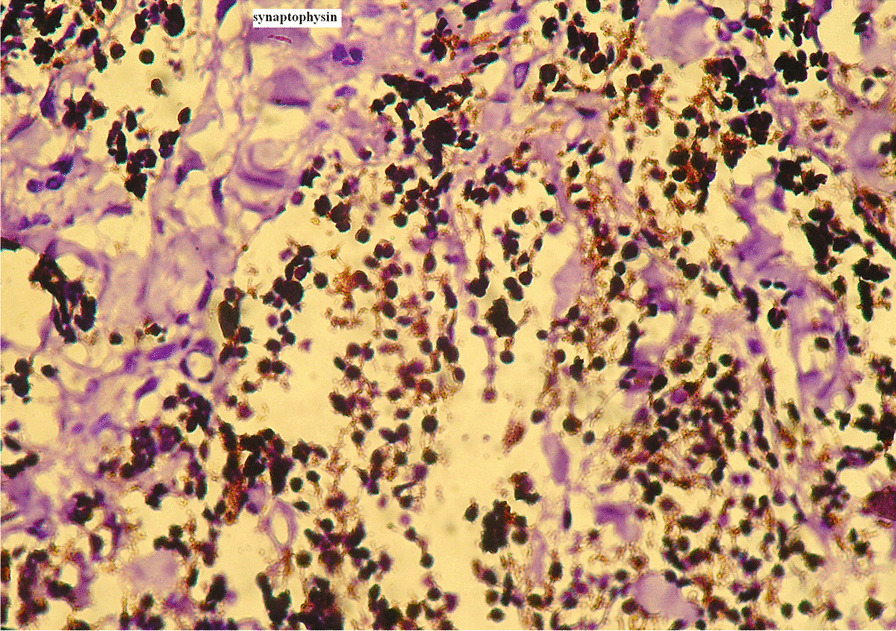
Synaptophysin positivity in the small round cells (dark brown)

Histopathologic examination of the excised tissue revealed the characteristic biphasic pattern of cell distribution: (1) smaller round cells with scanty cytoplasm and hyperchromatic nuclei in a fibrillary & vascularized fibrocollagenous background (Fig. 2A–C) and (2) cell population consisting of larger cells with vesicular nuclei and eosinophilic cytoplasm containing brownish pigment which cluster in alveolar and pseudoglandular patterns (Fig. 2D, E). Immunohistochemical confirmation of the tumor for neural origin in our case was also done by synaptophysin and chromogranin with control. The small round cells stained strongly for synaptophysin, except few focal areas (Fig. 3), and weakly stained for chromogranin. The pigmented larger cells remained pigmented in both cases. No anaplasia or mitosis was noted. This histopathologic examination of the tissue confirmed the diagnosis of MNTI. The postoperative course was uneventful. Three weeks after surgery, the patient was reevaluated and no re-growth was noted. The patient had a follow-up subsequently with no recurrence at the 6th and 9th months of visits.

## Discussion

Melanotic Neuroectodermal Tumor of Infancy, also named Pigmented Neuroectodermal Tumor of Infancy, is a rare, fast-growing, locally aggressive, clinically benign neoplasm of neuroectodermal origin [3–5]. It was first described by Krompecher in 1918 as ‘congenital Melanocarcinoma’ thereafter different names were used till the term MNTI was coined by Borello and Gorlin after confirmation of the nature & neuroectodermal origin of the tumor by immunocytochemistry & electromicroscopic study [2, 4, 6]. To date, a few hundred cases have been reported in worldwide literature.

The tumor commonly occurs in ages less than one year characteristically in infants of an average age of five months, with no variation in sex, predominantly in the maxillary alveolar process(~ 70%) as a cystic mass imposing difficulty in making a distinction from dermoid cyst radiographically [2, 7, 8]. In tumors occurring on the skull, plain radiographs may show adjacent bone sclerosis and hyperostosis [8]. Other less commonly reported sites of occurrence include the skull (~ 10.6%), especially the temporal area, and very few cases on the anterior fontanelle area, mandible, genital organs, and lower extremities [7–9]. MNTI is very rarely described in adolescents [10].

The neoplasm is histologically characterized by the presence of two groups of cells in a vascularized fibrocollagenous stroma. Small round cells (neuroblast-like) in sheets and small nests and larger cells with moderate to abundant cytoplasm containing coarse dark-brown pigment which represents a CNS-type melanin according to molecular studies on similar neoplasms. These cells frequently form pseudoglandular and alveolar spaces which are characteristic of this tumor and very useful to rule out other tumors like neuroblastoma and alveolar rhabdomyosarcoma [8, 11].

MNTI is known to recur with a reported risk of recurrence reaching 10 to 15%, especially in those where the tumor most probably was not excised completely [1]. Most recurrences occurred within the first or second month after primary surgical intervention because of incomplete (retrospectively) first resection [12]. Recurrence is reported after 3 months of the initial surgery in orbital MNTI in one case and after 4 months of the initial resection in mandibular tumor in another case [12, 13]. There is a similar description of MNTI on the anterior fontanelle with no recurrence after 10 months of excision [14]. However, a follow-up of at least two years is recommended by some authors based on the cumulative findings in tumors occurring on the skull [15]. A primary malignant MNTI is very rare and is usually reported as a component of immature teratoma and in tumors occurring in the thigh [7]. Rare cases of skull tumors pursuing a malignant course with secondary brain invasion are described [8].

MNTI being a rare lesion poses a challenge to clinicians not only in its diagnosis but also in management. Total excision including up to 1 cm free margin is recommended without adjuvant chemotherapy or radiotherapy by most authors to achieve a cure [2].

To the best of our knowledge, this kind of tumor is the first to be reported from Ethiopia. While the most common site of the tumor is the maxillary area, the tumor in this case was on the midline of the frontal skull. The classic histologic findings and the supportive positive immunohistochemistry tests for synaptophysin and chromogranin confirm the diagnosis. The clinical and radiographic findings are also compatible with findings in other reported cases in the skull and other locations. The absence of re-growth on follow-up foretells indeed the benign nature of the tumor in this case.

## Conclusion

Melanotic neuroectodermal tumor of infancy should be suspected by clinicians in infants presenting with head and neck tumors. A high index of suspicion is required to diagnose this tumor and should be differentiated from other round-cell tumors. Wide-margin excision is associated with a cure, but patients should be followed for possible recurrence and rare malignant transformation.

## Data Availability

Not applicable.
